# Feasibility of clinical performance assessment of medical students on a virtual sub-internship in the United States

**DOI:** 10.3352/jeehp.2021.18.12

**Published:** 2021-06-22

**Authors:** John Woller, Sean Tackett, Ariella Apfel, Janet Record, Danelle Cayea, Shannon Walker, Amit Pahwa

**Affiliations:** 1 Department of Medicine, Johns Hopkins Hospital, Baltimore, MD, USA; 2 Rosebud Hospital, Rosebud, SD, USA; Hallym University, Korea

**Keywords:** Medical student, Undergraduate medical education, Distance education, Clinical clerkship

## Abstract

We aimed to determine whether it was feasible to assess medical students as they completed a virtual sub-internship. Six students (out of 31 who completed an in-person sub-internship) participated in a 2-week virtual sub-internship, caring for patients remotely. Residents and attendings assessed those 6 students in 15 domains using the same assessment measures from the in-person sub-internship. Raters marked “unable to assess” in 75/390 responses (19%) for the virtual sub-internship versus 88/3,405 (2.6%) for the in-person sub-internship (P=0.01), most frequently for the virtual sub-internship in the domains of the physical examination (21, 81%), rapport with patients (18, 69%), and compassion (11, 42%). Students received complete assessments in most areas. Scores were higher for the in-person than the virtual sub-internship (4.67 vs. 4.45, P<0.01) for students who completed both. Students uniformly rated the virtual clerkship positively. Students can be assessed in many domains in the context of a virtual sub-internship.

## Background/rationale

Medical students acquire clinical skills while caring for patients under the supervision of residents and attending physicians. Assessments from supervisors form a component of students’ grades; while it is recommended to deliver assessments based on direct observations of clinical skills, doing so can be challenging in the busy context of clinical care. Therefore, supervisors often rely on indirect observations of clinical skills through students’ prepared oral presentations and documentation [1].

Due to the coronavirus disease 2019 (COVID-19) pandemic, the American Association of Medical Colleges (AAMC) recommended in March 2020 that medical schools immediately suspend clinical rotations. Medical school administrators looked for ways to fill this gap. Some institutions created ways to integrate medical students into clinical work, even though they were not physically present in the hospital [2-8]. With the incorporation of video conferencing platforms into patient care, some students cared for patients virtually, including remote observation and communication with patients and family members [9]. However, little is known about the assessment of medical students’ remote participation in medical care.

## Objectives

This study aimed to determine the feasibility of assessing students on a virtual clerkship. Specifically, the clinical performance of medical students who participated in both an in-person sub-internship and virtual sub-internship was assessed using the same measurement tool.

## Ethics statement

This study was approved by the Johns Hopkins Medicine Institutional Review Board (IRB00267680). The requirement to obtain informed consent was waived by the Institutional Review Board.

## Study design

This is a report-based comparison study.

## Participants

This study included students who had completed an in-person sub-internship during the 2020 calendar year, some of whom also completed a virtual sub-internship for 2 weeks from April to June 2020. The in-person sub-internship was 3 weeks long during this academic year.

## Setting

**Fig. 1.**
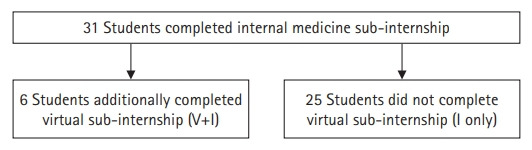
Students included in the study. V+I, virtual and in-person; I only, those who only completed in-person.

We created a model for a virtual sub-internship in internal medicine at the Johns Hopkins Hospital (JHH) and Johns Hopkins Bayview Medical Center (JHBMC) in which students would virtually join the same teams as on in-person rotations. From April to June 2020, fourth-year medical students at Johns Hopkins University School of Medicine participated in a remote virtual sub-internship for 2 weeks. This elective was graded on a pass/fail basis. Students joined rounds (including bedside rounds) using video-conferencing applications and portable tablet-style computers. Students called patients and their families to conduct interviews and share health information. Communication with the on-site team utilized telephone, video conference, and Health Insurance Portability and Accountability Act–compliant messaging applications. Students were included in our analysis if they completed the in-person sub-internship during the 2020 calendar year; a subset of this group of students also completed the virtual sub-internship in 2020 (Fig. 1).

## Data source/measurement

Upon completion of the virtual sub-internship, residents and faculty documented their assessments of student performance using the same clinical performance assessment (CPA) that was used for the in-person rotation and across the institution for all clinical clerkships. For the CPA, supervisors rate students along 15 clinical domains, each measured by items with 5 unique descriptive anchors, corresponding to numeric values of 1–5, with 5 being the most favorable. Evaluators had the option to respond “unable to assess” for each domain (as on the in-person CPA) (Supplement 1). We asked evaluators whether assessing students in the virtual context was harder, easier, or equally difficult in each domain (Supplement 1). The CPA used in this paper to evaluate students is not externally validated. It is based on the Core Competencies as described by the Accreditation Council for Graduate Medical Education (ACGME), and is used for all medical students on clinical clerkships at the Johns Hopkins University School of Medicine. Therefore, while this CPA is widely used, data supporting its external validation are not available, to our knowledge. Students completed an evaluation of the clerkship, in which they were asked to rate the overall virtual sub-internship and the quality of their experiences in specific activities when compared to in-person clerkships (Supplement 2). The reliability of the measurement tool for students’ evaluation of the clerkship was not tested due to the complexity of its items and the low number of subjects.

## Variables

The variables were student ratings by supervisors and questionnaire responses by students and supervisors.

## Bias

Students self-selected to complete the virtual sub-internship and were not assigned or chosen.

## Study size

The study size was not pre-determined; instead, it was based on the number of students who chose to complete the virtual sub-internship.

## Statistical methods

We recorded the frequency with which evaluators were unable to assess students in each domain on the virtual and in-person clerkship. We used the paired 2-tailed t-test to compare the overall frequency of “unable to assess” responses between the virtual and in-person sub-internships, and the Fisher exact test to compare “unable to assess” responses in each domain. We compared assessments using the CPA between the virtual sub-internship and the in-person sub-internship for students who completed both as composite scores and in each domain of assessment. We also compared CPA results from the in-person sub-internship for those who completed both sub-internships (virtual and in-person, V+I) to those who only completed in-person (I only). Because students had assessments completed by multiple supervisors but did not have the same number of assessments, we used a generalized estimating equation to compare student group data. The specific GEE model used was a proportional odds model to compare the odds of receiving a higher score between groups for each domain. The data presented in this paper were analyzed using SAS ver. 9.4 (SAS Institute Inc., Cary, NC, USA).

## Comparison of students’ performance between the in-person sub-internship and virtual sub-internship

In the 2020 calendar year, 31 students completed the in-person sub-internship at JHH or JHBMC. Six of these students additionally completed the virtual sub-internship prior to their in-person sub-internship. All students who completed the virtual sub-internship subsequently completed the in-person sub-internship.

Students received completed CPAs from an average of 5.6 resident or attending evaluators (range, 2–11) on the in-person sub-internship, and an average of 4.3 (range, 2–7) on the virtual sub-internship. Twenty-six raters completed CPAs for 6 students on the virtual sub-internship; since each student was able to be evaluated in 15 domains, there were 390 possible items for assessment. Raters marked “unable to assess” 75 times (19%), most frequently in the domains of the physical examination (21/26, 81%), rapport with patients (18/26, 69%), and compassion (11/26, 42%) (Table 1, Dataset 1). Excluding these three domains, raters responded “unable to assess” 25 of 312 times (8%). By comparison, in the 227 completed CPAs from the in-person sub-internship, out of 3,405 items, raters responded “unable to assess” 88 times (2.6%), reflecting a statistically meaningful difference (P=0.01). Of these 88 responses, 44 (19% of raters) were in the domain of the physical examination.

Students who previously completed the virtual sub-internship (V+I), compared with those who did not (I only), received numerically higher scores in 18 of 20 domains, but only 2 domains showed statistically significantly higher scores: basic science knowledge (4.66 versus 4.42, P<0.01) and responsibility/reliability (4.88 versus 4.77, P=0.02). The overall scores did not significantly differ between these groups.

## Ease of assessing student performance remotely, as reported by raters

When asked whether it was easier, equally difficult, or more difficult to assess students in the virtual environment, 21 raters responded, yielding a total of 253 responses. Overall, 128 responses (51%) indicated assessing students was equally difficult, 119 (47%) indicated it was harder in the virtual clerkship, and 6 (2%) indicated that it was easier. All 19 raters (100%) who responded to the corresponding question indicated that the physical examination was harder to assess remotely (Table 1, Dataset 1).

## Medical students’ evaluation on the virtual sub-internship

In a comparison of the assessments of the 6 students who completed both virtual and in-person sub-internships (V+I) from the virtual sub-internship to their assessments from the in-person sub-internship, the overall scores (combining scores in all areas of assessment) were higher in the in-person sub-internship than in the virtual sub-internship (4.67 versus 4.45, P<0.01). Significantly higher scores were found in the individual domains of basic science knowledge (4.66 versus 3.92, P<0.01), clinical knowledge (4.65 versus 4.27, P<0.01), self-directed learning (4.73 versus 4.42, P=0.04), and responsibility/reliability (4.88 versus 4.62, P<0.01). The other domains did not differ significantly (Table 2, Dataset 1).

When students evaluated the virtual sub-internship, all reported it was “excellent” or “outstanding.” Students were asked to compare various components of the rotation with in-person rotations. The responses were mixed, but overall, 24 of 36 responses (67%) indicated that the activities in the virtual clerkship were the same or better.

## Key results

This study shows that it is feasible to assess many domains of clinical skills in a virtual clerkship. Most students received assessments from their raters in all domains, with only a few domains presenting challenges: raters most frequently responded “unable to assess” in domains of the physical examination, rapport with patients, and compassion. More than half of raters also indicated it was harder to assess students in the remote context in the domains of clinical knowledge, history-taking skills, and respectfulness.

## Interpretation

Interestingly, the domain of recording patient data also had more frequent “unable to assess” responses (although the numerical difference was small), although only 7% of respondents stated that it was harder to assess this domain remotely. This may reveal a discordance between raters’ perception of difficulty and their ability to rate students, or it may be the result of the small sample size. In 8 of 15 domains, raters were no more likely to respond “unable to assess” on the virtual than the in-person clerkship, including critical areas such as basic science knowledge, clinical knowledge, responsibility, rapport with colleagues, and oral patient presentations. Future virtual clerkships may benefit from specific means of addressing the areas in which students were not reliably assessed: examples include objective structured clinical examinations to assess physical examination skills or structured patient queries to assess student rapport with patients.

Forty-four raters (19%) also marked “unable to assess” for physical examination skills on the in-person sub-internship, far higher than any other domain. Even in the in-person environment, students do not always receive close observation at the patient’s bedside to determine their grade. The virtual clerkship may therefore not be as different, in terms of student assessment, as we might assume.

## Limitations

This is a single-institution study with a small number of subjects. Therefore, it is difficult to generalize the results to other institutions or countries.

## Conclusion

While virtual clerkships may not replace in-person clerkships, this report shows that a virtual clerkship can engage students in patient care and provide a means of assessment without requiring students to be physically present in the hospital. Students rated the overall sub-internship favorably, and although some areas were more challenging in the remote environment, students felt engaged with the medical team and patients. This curriculum can provide a means for continued clinical learning when there are restrictions on how students can see patients due to the COVID-19 pandemic. Additionally, remote clerkships may be useful in situations wherein students are unable to travel to other locations (due to cost or other barriers). Examples could include students seeking clinical experience in a different region or country; students seeking exposure to specialties not offered at their institution; or students exploring a department or specialty at an institution where they may apply for residency without the financial and time cost associated with away rotations.

## Figures and Tables

**Table 1.** Evaluators’ responses regarding difficulty assessing students in the remote versus in-person sub-internship

Domain	Evaluators^a),b)^ responding “unable to assess” on virtual clerkship	Evaluators^a),c)^ responding “unable to assess” on in-person clerkship	P-value^d)^	Evaluators^a)^ who felt evaluation was more difficult in virtual context than in-person
Basic science knowledge	2 (8)	5 (2)	0.15	8 (40)
Clinical knowledge	0	0	1	12 (57)
Self-directed learning	1 (4)	0	0.1	2 (11)
History taking skills	8 (31)	7 (3)	<0.01^*^	10 (56)
Physical/mental status exam skills	21 (81)	44 (19)	<0.01^*^	19 (100)
Problem solving	2 (8)	3 (1)	0.08	8 (44)
Clinical judgment	1 (4)	1 (0.4)	0.19	6 (35)
Responsibility/reliability	0	0	1	4 (22)
Compassion	11 (42)	4 (2)	<0.01^*^	15 (88)
Respectfulness	3 (12)	2 (1)	<0.01^*^	8 (57)
Response to feedback	4 (15)	10 (4)	0.04^*^	4 (13)
Rapport with patients	18 (69)	11 (5)	<0.01^*^	12 (92)
Rapport with colleagues	1 (4)	0	0.1	7 (47)
Oral patient presentations	1 (4)	0	0.1	3 (20)
Recording clinical data	2 (8)	1 (0.4)	0.01^*^	1 (7)

Values are presented as number (%).

a) Residents or attendings who supervised the sub-intern and completed the Clinical Performance Assessment tool for the student.

b) Out of 26 evaluators.

c) Out of 227 evaluators.

d) The Fisher exact test was used to compare the frequency of “unable to assess” responses between virtual and in-person clerkships.

* Denotes a statistically significant difference.

**Table 2.** Comparison of scores from the virtual (V) versus in-person (V+I) sub-internship for the 6 students who completed both (whether V+I was more likely to have a higher score than V)

Domain	Odds ratio of the V+I mean score being higher than the V score (95% confidence interval)	P-value
Basic science knowledge	3.93 (1.91–8.05)^*^	<0.01
Clinical knowledge	3.59 (1.43–9.04)^*^	<0.01
Self-directed learning	1.81 (1.02–3.21)^*^	0.04
History taking skills	3.04 (0.86–10.76)	0.08
Physical/mental status exam skills	2.25 (0.51–9.92)	0.3
Problem solving	2.20 (0.96–5.01)	0.06
Clinical judgment	1.83 (0.73–4.59)	0.19
Responsibility/reliability	5.01 (2.32–10.85)^*^	<0.01
Compassion	1.89 (0.66–5.41)	0.23
Respectfulness	1.25 (0.21–7.49)	0.81
Response to feedback	1.55 (0.36–6.72)	0.56
Rapport with patients	1.59 (0.64–3.92)	0.32
Rapport with colleagues	2.73 (0.53–14.14)	0.23
Oral patient presentations	1.29 (0.76–2.21)	0.34
Recording clinical data	1.64 (0.73–3.7)	0.24

* Denotes a statistically significant difference.

## Footnotes

**Data availability** Data files are available from Harvard Dataverse: https://doi.org/10.7910/DVN/BA06HE Dataset 1. Raw response data from 31 participating medical students and 20 raters of the sub-internship of Johns Hopkins University in 2020.

## Supplementary materials

Data files are available from Harvard Dataverse: https://doi.org/10.7910/DVN/BA06HE

Supplement 1. Measurement tool for the clinical performance assessment used by raters and students.

Supplement 2. Questionnaire for 6 medical students for their evaluations on the virtual sub-internship.

Supplement 3. Audio recording of the abstract.
