# Supplementary material for: Feasibility of clinical performance assessment of medical students on a virtual sub-internship in the United States
Source: J Educ Eval Health Prof. 2021 Jun 22;18:12. doi: 10.3352/jeehp.2021.18.12 (PMC8289686; doi:10.3352/jeehp.2021.18.12)
Supplement: Supplementary file 2 — Supplement 1. Measurement tool for the clinical performance assessment used by raters and students. [file jeehp-18-12-suppl1.pdf]

Basic Information

Thank you for supervising a student during our pilot of virtual rounding in the COVID-19 era. We would like your feedback on how we can assess students during a remote inpatient learning experience.

Most of what follows is our typical sub-internship form. Please complete it as you usually would. We have also included space for you to comment on each item about how validly it could be assessed during student remote participation.

Medical Student Name

Evaluator Name, Role

Please answer the following questions for this remote elective pilot.

It was easy to incorporate a medical student into the ward team remotely.

Strongly disagree	Somewhat disagree	Neither agree nor disagree	Somewhat agree	Strongly agree
○	○	○	○	○

Compared to a traditional rotation, the amount of work I had to perform for a patient with a student assigned to him/her on this elective:

Decreased	Remained the same	Increased
○	○	○

In the future, I would have a student participate remotely in patient care.

Strongly disagree	Somewhat disagree	Neither agree nor disagree	Somewhat agree	Strongly agree
○	○	○	○	○

Block 1

Please provide a rating. Then provide comments on how valid you feel your assessment was in this pilot, with the student participating from home, vs a traditional rotation, where the student would be present in the hospital each day.

Note that while you may not have directly observed a student performing a task you may be able to assess their ability to perform a task based on their oral presentation (e.g. You can assess their physical exam skills but how they describe the physical exam in the oral presentation).

Basic Science Knowledge

- ○ Not Observed
- ○ Critical Deficiencies - Major deficiencies in knowledge base. Consistently inaccurate
- ○ Often inaccurate. Incomplete understanding of basic concepts.
- ○ Sound understanding of pathologic substrate of important clinical disorders

- Thorough textbook understanding of pathophysiology and mechanisms of therapeutic interventions.
- Aware of current concepts of disease pathogensis and therapeutic mechanisms of action. Asks probing scientific questions at the 'cutting edge'.

Referring to the previous question, comment on how your ability to assess was influenced by remote participation (e.g., harder, unaffected, easier)?

Clinical Knowledge

- Not observed
- Critical deficiencies. Major deficiencies in knowledge base. Consistently inaccurate.
- Inconsistent understanding of patient problems. Limited differential diagnosis.
- Knows basic differential diagnoses of major/active problems in patients. Understands team's choice of therapy.
- Knows expanded differential diagnoses, including recognition of emergencies. Can discuss therapeutic options.
- Knows nuances of differential diagnosis, including disease prevalence and anticipated history & exam findings. Able to independently formulate a management plan. Able to assign prognoses.

Referring to the previous question, comment on how your ability to assess was influenced by remote participation (e.g., harder, unaffected, easier)?

Self Directed Learning

- ○ Not observed
- ○ Critical deficiencies. No self directed learning. Lack of effort. Denies inadequacies.
- ○ Rarely self directed. Frequent prompting required. Minimal evidence of outside work.
- ○ Appropriate use of textbooks and reference sources. Well prepared. Clearly motivated.
- ○ Sets goals. Reads in advance.Timely use of resources. Consistent effort to improve.
- ○ Never needs prompting. Outstanding initiative. Effective use of resources. Educates others.

Referring to the previous question, comment on how your ability to assess was influenced by remote participation

(e.g., harder, unaffected, easier)?

Data Gathering (History/Interviewing)

- Not observed
- Critical deficiencies. Disorganized. Fails to gather important historical information or patients perspective. Fails to identify patients agenda. Never reviews old records or seeks collateral informants.
- Incomplete at times. Frequent closed-ended questioning. Unfocused. Inappropriate communications rendered. Rarely reviews old records.
- Obtains appropriate basic history in patient-centered manner. May need attention to efficiency and exploration of difficult ideas. Reviews old records as available.
- Precise, detailed history appropriate to the setting. Seeks understanding of patient perspective. Seeks collateral informants when appropriate.
- Consistently precise and efficient detailed history of illness. Insightful and consistently elicits subtle historical points. Gathers and carefully reviews old records.

Referring to the previous question, comment on how your ability to assess was influenced by remote participation (e.g., harder, unaffected, easier)?

Physical/ Mental Status Exams

- ○ Not observed
- ○ Critical deficiencies. Unreliable, incomplete, disorganized. Major deficiencies. Inadequate technique. Disregards patient comfort.
- ○ Sometimes unreliable, incomplete, or disorganized. Some major findings missed. Exam skills need refinement.
- ○ Most findings identified. Usually logical and focused exam. Properly structured, properly ordered, few omissions, considers patients comfort.
- ○ Very comfortable approach to the exam. Major findings always identified. Focused, organized skills.
- ○ Well organized. Subtle findings elicited. Very efficient.

Referring to the previous question, comment on how your ability to assess was influenced by remote participation (e.g., harder, unaffected, easier)?

Problem Solving

- ○ Not observed
- ○ Critical deficiencies. Cannot interpret basic data. Unable to identify basic issues on problem list.
- ○ Frequently reports data without interpretation. May be able to list problems, but analysis of issues is superficial. Cannot prioritize problems.
- ○ Constructs appropriate problem lists and develops basic differential diagnosis. Analysis of problems reflects basic understanding of issues.
- ○ Consistently offers reasonable interpretation of data. Arrives at prioritized differential diagnosis.
- ○ Consistently understands complex issues and interrelated patient problems. Always well prioritized.

Referring to the previous question, comment on how your ability to assess was influenced by remote participation (e.g., harder, unaffected, easier)?

Clinical Judgment

- ○ Not observed

- ○ Critical deficiencies. Consistently poor judgment. Actions may affect patients adversely.
- ○ Inconsistent understanding of patient problems. Inability to identify critical nature of illness.
- ○ Can recognize priorities in clinical situations. Aware of own limitations. Able to recognize a sick patient.
- ○ Weighs pros and cons of diagnostic plans. Incorporates patient perspectives. Prioritizes well.
- ○ Insightful approach to management plans. Anticipates changes. Appreciates management subtleties.

Referring to the previous question, comment on how your ability to assess was influenced by remote participation (e.g., harder, unaffected, easier)?

Responsibility/ Reliability

- ○ Not observed
- ○ Critical deficiencies. Unexplained absences. Unreliable. Inappropriately dressed. Does not follow through with assigned tasks.
- ○ Often unprepared, lackadaisical, needs reminders in fulfillment of responsibilities. Often late, appears uninterested.
- ○ Punctual. Fulfills assigned tasks consistently. Responsible and reliable.

- ○ Seeks and is capable of taking on additional responsibility.
- ○ Excellent initiative. Unusual dedication to patient care and education.

Referring to the previous question, comment on how your ability to assess was influenced by remote participation (e.g., harder, unaffected, easier)?

Compassion

- ○ Not observed
- ○ Critical deficiencies. Consistently uncaring and insensitive. Seemingly unaware of patient suffering and needs.
- ○ Often misses patients cues of distress, suffering. Only occasionally offers encouragement.
- ○ Regularly recognizes and responds to patient distress appropriately. Sympathetic, develops rapport.
- ○ Consistently aware of patients suffering and needs. Clearly warm and caring. Gains confidence and trust.
- ○ Actively addressed emotional implications of illness with patient and family.

Referring to the previous question, comment on how your ability to assess was influenced by remote participation (e.g., harder, unaffected, easier)?

Respectfulness

- Not observed
- Critical deficiencies. Inconsiderate, lacks respect. Unable to work with medical team. Indifferent to concerns/opinions of others.
- Occasional lapses in respectful behavior. Often intolerant to opinions/concerns of others.
- Considerate and shows appropriate deference. Treats patients, families, and staff with respect. Culturally sensitive.
- Shows respect in difficult situations such as conflict resolution. Extra effort in cultural sensitivity.
- Actions can both foster and maintain respectful environment.

Referring to the previous question, comment on how your ability to assess was influenced by remote participation (e.g., harder, unaffected, easier)?

Response to Feedback

- ○ Not observed
- ○ Critical deficiencies. Completely unaware of own inadequacies. Uninterested in receiving feedback on performance. Refuses or makes no effort to improve.
- ○ Resistive or defensive in accepting feedback. Makes marginal efforts to improve with feedback.
- ○ Accepts and generally makes effort to improve with feedback.
- ○ Seeks and accepts feedback. Shows consistent improvement.
- ○ Able to self assess and make necessary changes for growth and progress.

Referring to the previous question, comment on how your ability to assess was influenced by remote participation (e.g., harder, unaffected, easier)?

Rapport with Patients

- ○ Not observed
- ○ Critical deficiencies. Avoids contact with patients. Little attempt to establish therapeutic rapport relationship. Disorganized. Generally unable to communicate facts to patients.
- ○ Difficulty establishing physician-patient relationships. Insensitive to patient needs.
- ○ Good skills at developing rapport with patients and responding to emotional needs of patients.
- ○ Excellent communication skills. Able to gain trust and confidence of patients and families.
- ○ Student becomes preferred contact for patients due to highly effective communication.

Referring to the previous question, comment on how your ability to assess was influenced by remote participation (e.g., harder, unaffected, easier)?

Rapport with Colleagues

- ○ Not Observed
- ○ Critical deficiencies. Avoids contact with team members. Inadequate skills to establish relationships with medical colleagues and other health professionals.
- ○ Sometimes has difficulty in relating well to medical colleagues and other health professionals. Maintains distance from team.
- ○ Relates well with medical colleagues and other health professionals. "Good team player." Functions well within the team structure.
- ○ Able to establish excellent rapport with medical colleagues and other health professionals. Gains confidence of team leaders.
- ○ Establishes tone of mutual respect and dignity with medical colleagues and other health professionals. Highly integrated into the team structure.

Referring to the previous question, comment on how your ability to assess was influenced by remote participation (e.g., harder, unaffected, easier)?

Oral Patient Presentations

- ○ Not observed
- ○ Critical deficiencies. Unprepared. Major omissions frequent. Poorly organized and incomplete. Misses key data.

- Presentations incomplete. Poorly focused. Some omissions in characterizations, chronology and pertinent data. Irrelevant facts. Rambling. Relies heavily on notes.
- Uses proper format. Acceptable delineation of primary problems with reasonable characterization and chronology. Culturally-sensitive presentations. Minimal use of notes.
- Presentation usually complete, well characterized and organized. May need attention to length and efficiency, and summarization of problems and plans.
- Consistently clear and succinct delineation of patient story. Accurate characterization with differential diagnosis and plan. Mastery of oral presentations.

Referring to the previous question, comment on how your ability to assess was influenced by remote participation (e.g., harder, unaffected, easier)?

Recording Clinical Data

- Not observed
- Critical deficiencies. Disorganized, incomplete, incoherent flow to written work. Illegible. Always late.

- ○ Often lacks relevant details. Somewhat disorganized. Often late. Occasional omissions in problems list.
- ○ Usually organized and complete. Adequate problem list/formulation documented. Timely.
- ○ Concise, well organized, comprehensive.
- ○ Superior organization. Sophisticated problem list/formulation and diagnostic/therapeutic approach. Documentation reflects thorough understanding of problems/ formulation.

Referring to the previous question, comment on how your ability to assess was influenced by remote participation (e.g., harder, unaffected, easier)?

Integrity

- ○ Unreliable, Lapses in: truthfulness, honesty with patients and peers, respect for other health professionals, patients or families. Disregards need for self-assessment.
- ○ Appropriate behavior of honesty, truthfulness and respect.

Please provide a narrative assessment of the student's performance.

Compare and contrast your ability to assess a student on this elective versus an in-person clinical rotation.

Feel free to share any additional thoughts you have.

Prepardness

Powered by Qualtrics
