# Supplementary material for: Feasibility of clinical performance assessment of medical students on a virtual sub-internship in the United States
Source: J Educ Eval Health Prof. 2021 Jun 22;18:12. doi: 10.3352/jeehp.2021.18.12 (PMC8289686; doi:10.3352/jeehp.2021.18.12)
Supplement: Supplementary file 3 — Supplement 2. Questionnaire for 6 medical students for their evaluations on the virtual sub-internship. [file jeehp-18-12-suppl2.pdf]

Default Question Block

Please rate your confidence for each of the following:

	No confidence	A little confident	Somewhat confident	Very confident	Completely confident	No[illegible] applic[illegible] (n[illegible] experi[illegible] to b[illegible] judgm[illegible]
Taking a history from a patient remotely						
Performing elements of physical exam remotely						
Communicating complex medical concepts with patients remotely						
Answering questions about a patient from his/her family remotely						
Conducting motivational interviewing with patients remotely						
Counseling patients after they were discharged						
Communicating with the hospital physician ward team remotely						
Communicating with others on the healthcare team remotely						

Rate the quality of the medicine tele-clerkship elective.

Poor	Fair	Good	Excellent	Outstanding

Compared to an in-person medicine rotation, please indicate if the following aspects were worse, the same, or better.

	Much worse	Somewhat worse	Same	Somewhat better	Much better
Oral presentations to the teams					
Engagement with the medical team					

	Much worse	Somewhat worse	Same	Somewhat better	Much better
Communicating with family about status of patient	○	○	○	○	○
Conducting motivational interviewing	○	○	○	○	○
Coordinating post-discharge care	○	○	○	○	○
Didactics	○	○	○	○	○

Compared to an in-person medicine rotation, what were the biggest challenges you faced during this elective?

What were the advantages to this remote clinical rotation, compared to an in-person medicine rotation

What surprised you about this elective?

What suggestions do you have for changes to make for future students participating in a remote clinical elective?

What worked especially well? What should we be sure not to change?

Please share any additional comments you'd like.

Powered by Qualtrics
